# Supplementary material for: Neutrophils restrain sepsis associated coagulopathy via extracellular vesicles carrying superoxide dismutase 2 in a murine model of lipopolysaccharide induced sepsis
Source: Nat Commun. 2022 Aug 6;13:4583. doi: 10.1038/s41467-022-32325-w (PMC9357088; doi:10.1038/s41467-022-32325-w)
Supplement: Supplementary file 1 — Supplementary Information [file 41467_2022_32325_MOESM1_ESM.pdf]

Supplementary information

Neutrophils restrain sepsis associated coagulopathy via extracellular vesicles carrying superoxide dismutase 2 in a murine model of lipopolysaccharide induced sepsis.

Wenjie Bao[1, *], Huayue Xing[1, *], Shiwei Cao[2], Xin Long[3], Haifeng Liu[1], Junwei Ma[1], Fan Guo[3, 4], Zimu Deng[1] and Xiaolong Liu[1, 2, 5, #]

[1] State Key Laboratory of Cell Biology, CAS Center for Excellence in Molecular Cell Science, Shanghai Institute of Biochemistry and Cell Biology, University of Chinese Academy of Sciences, Chinese Academy of Sciences, 320 Yueyang Road, Shanghai, China

[2] School of Life Science and Technology, Shanghai Tech University, Shanghai, 200031, China.

[3] State Key Laboratory of Stem Cell and Reproductive Biology, Institute of Zoology, Chinese Academy of Sciences, Beijing 100101, China

[4] Institute for Stem Cell and Regeneration, Chinese Academy of Sciences, Beijing 100101, China

[5] Key Laboratory of Systems Health Science of Zhejiang Province, School of Life Science, Hangzhou Institute for Advanced Study, University of Chinese Academy of Sciences, Hangzhou, 310024, China.

*These authors contributed equally

#Leading contact

Correspondence: dengzimu@sibcb.ac.cn; liux@sibcb.ac.cn

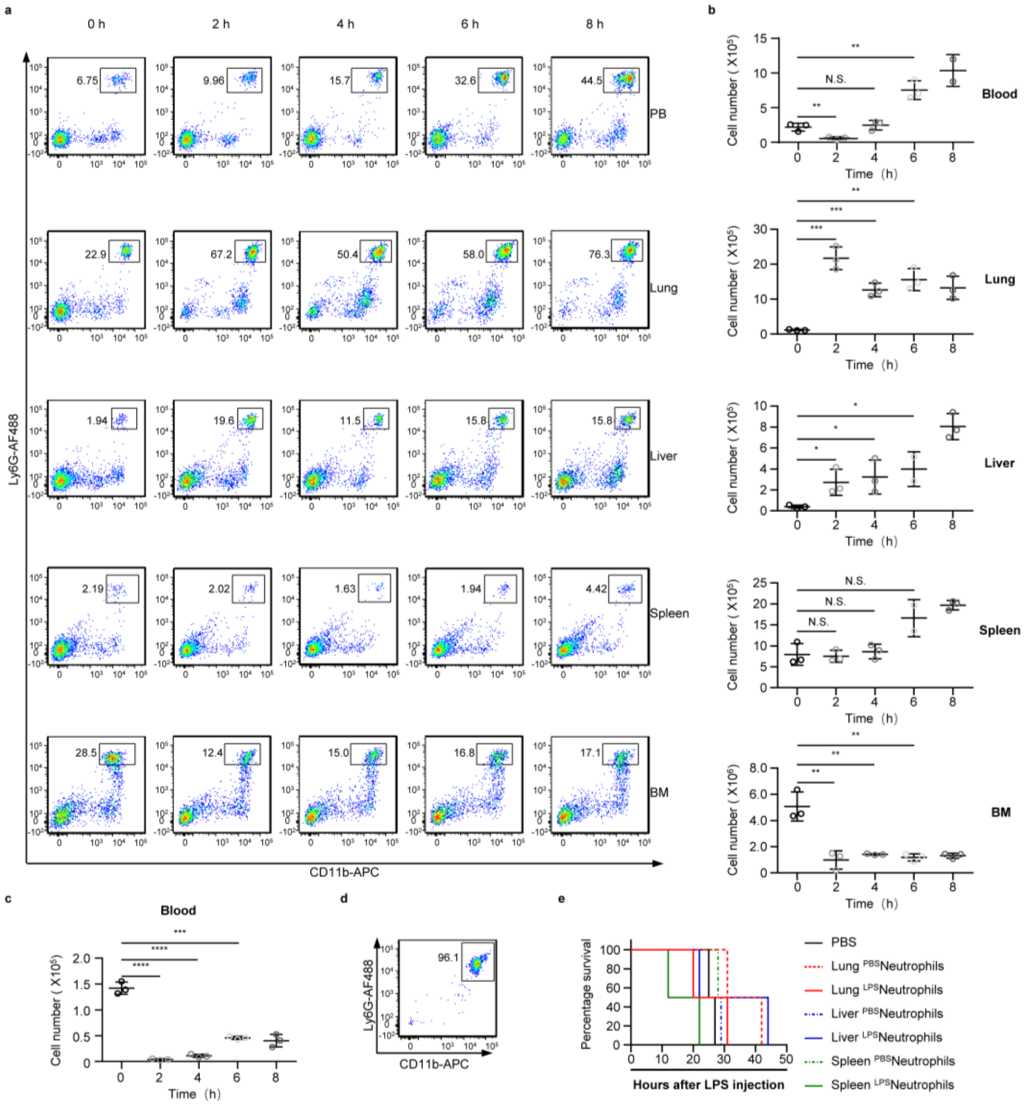

Supplementary Figure 1. Neutrophils migrate from bone marrow to different organs in response to LPS.

(**a**) Flow plots showing the expression of CD11b versus Ly6G among CD45^{+} cells after LPS.

(**b**) Total cell counts of neutrophils (CD11b^{+}, F4/80^{+}, CD45^{+}) in peripheral blood 0 hours (n = 3), 2 hours (n = 3), 4 hours (n = 3), 6 hours (n = 3), and 8 hours (n = 2) after LPS. Total cell counts of neutrophils (CD11b^{+}, F4/80^{+}, CD45^{+}) in lung 0 hours (n = 3), 2 hours (n = 3), 4 hours (n = 3), 6 hours (n = 3), and 8 hours (n = 3) after LPS. Total cell counts of neutrophils (CD11b^{+}, F4/80^{+}, CD45^{+}) in liver 0 hours (n = 3), 2 hours (n = 3), 4 hours (n = 3), 6 hours (n = 2), and 8 hours (n = 3) after LPS. Total cell counts of neutrophils (CD11b^{+}, F4/80^{+}, CD45^{+}) in spleen 0 hours (n = 3), 2 hours (n = 3), 4 hours (n = 3), 6 hours (n = 2), and 8 hours (n = 3) after LPS. Total cell counts of neutrophils

(CD11b$^+$, F4/80$^+$, CD45$^+$) in bone marrow 0 hours (n = 3), 2 hours (n = 3), 4 hours (n = 3), 6 hours (n = 3), and 8 hours (n = 3) after LPS.

(**c**) Total cell counts of macrophages (CD11b$^+$, F4/80$^+$, CD45$^+$) in peripheral blood after LPS (n = 3).

(**d**) The purity of negative selected neutrophils before transfer was checked by flow cytometry.

(**e**) Survival of mice after lethal LPS challenge. Mice were pre-transferred with LPS-primed neutrophils (^{LPS}neutrophils) from lung, liver or spleen, PBS-primed neutrophils (^{PBS}neutrophils) from lung, liver or spleen, or PBS only (n = 2 per group).

Source data are provided as a Source Data file. Data are mean ± *SD*. Two-tailed unpaired t tests were used for statistical analyses in (**b**) and (**c**). *$p < 0.05$, **$p < 0.01$, ***$p < 0.001$, ****$p < 0.0001$, N.S. not significant.

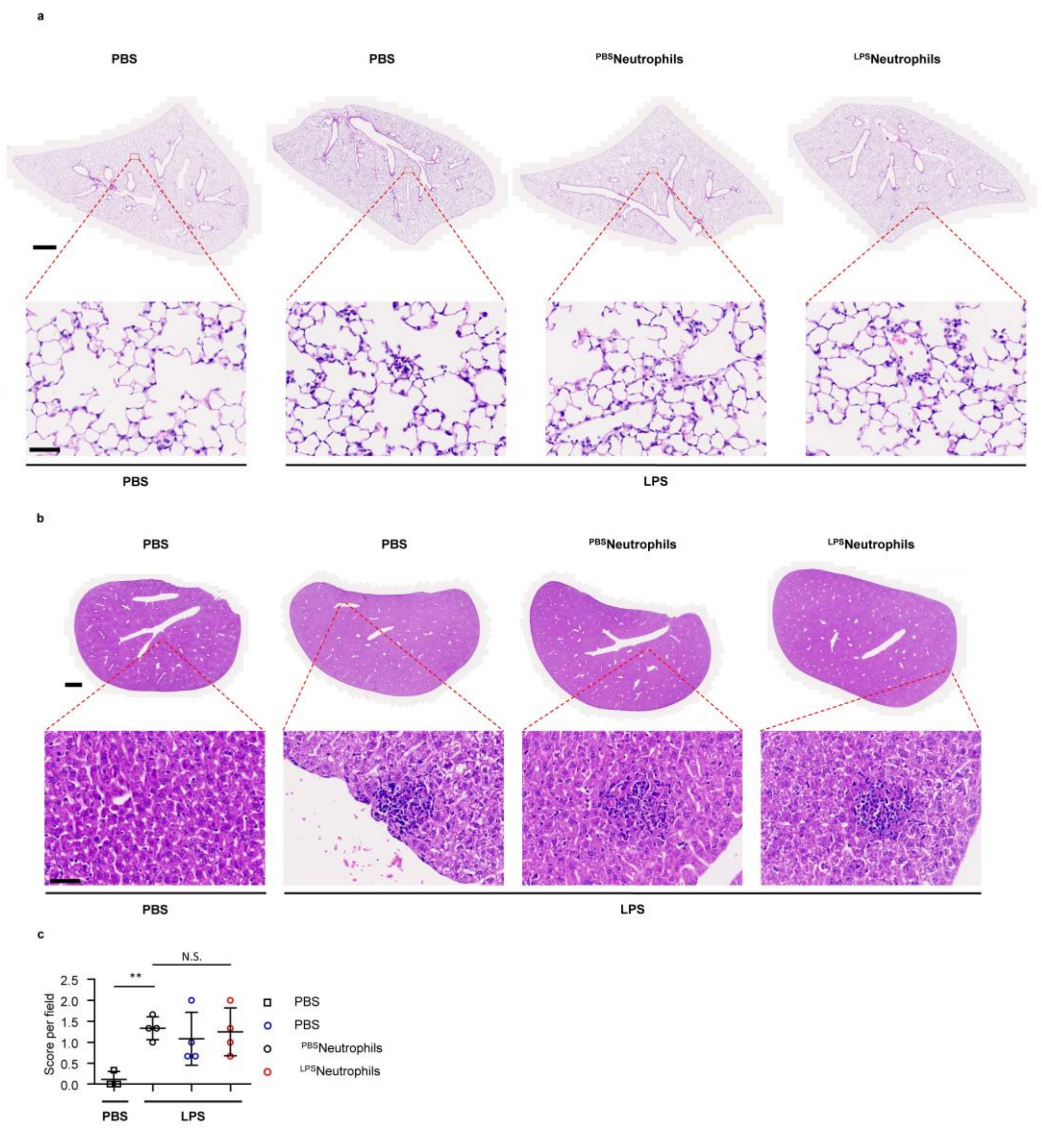

Supplementary Figure 2. LPS-primed neutrophils do not alter the initial immune infiltrations in tissues.

(a-b). Representative images of H&E staining of livers and lungs from the indicated recipient mice 1 hour after PBS or lethal LPS. Three independent replicate experiments were performed for each sample.

(**c**). Histology score of the H&E stainin We have stated how many times each experiment was repeated independently g of lung sections in mice after PBS (n = 3). Histology score of the H&E staining of lung sections in mice after lethal LPS. Mice were pre-transferred with LPS-primed PB neutrophils (^{LPS}neutrophils) (n = 4), PBS-primed PB neutrophils (^{PBS}neutrophils) (n = 4) or PBS only (n = 4).

Source data are provided as a Source Data file. Scale bar: 1000 μm in zoomed-out view and 50 μm in zoomed-in view. Data are mean ± *SD*. Two-tailed unpaired t tests were

used for statistical analyses.** $p < 0.01$, N.S. not significant.

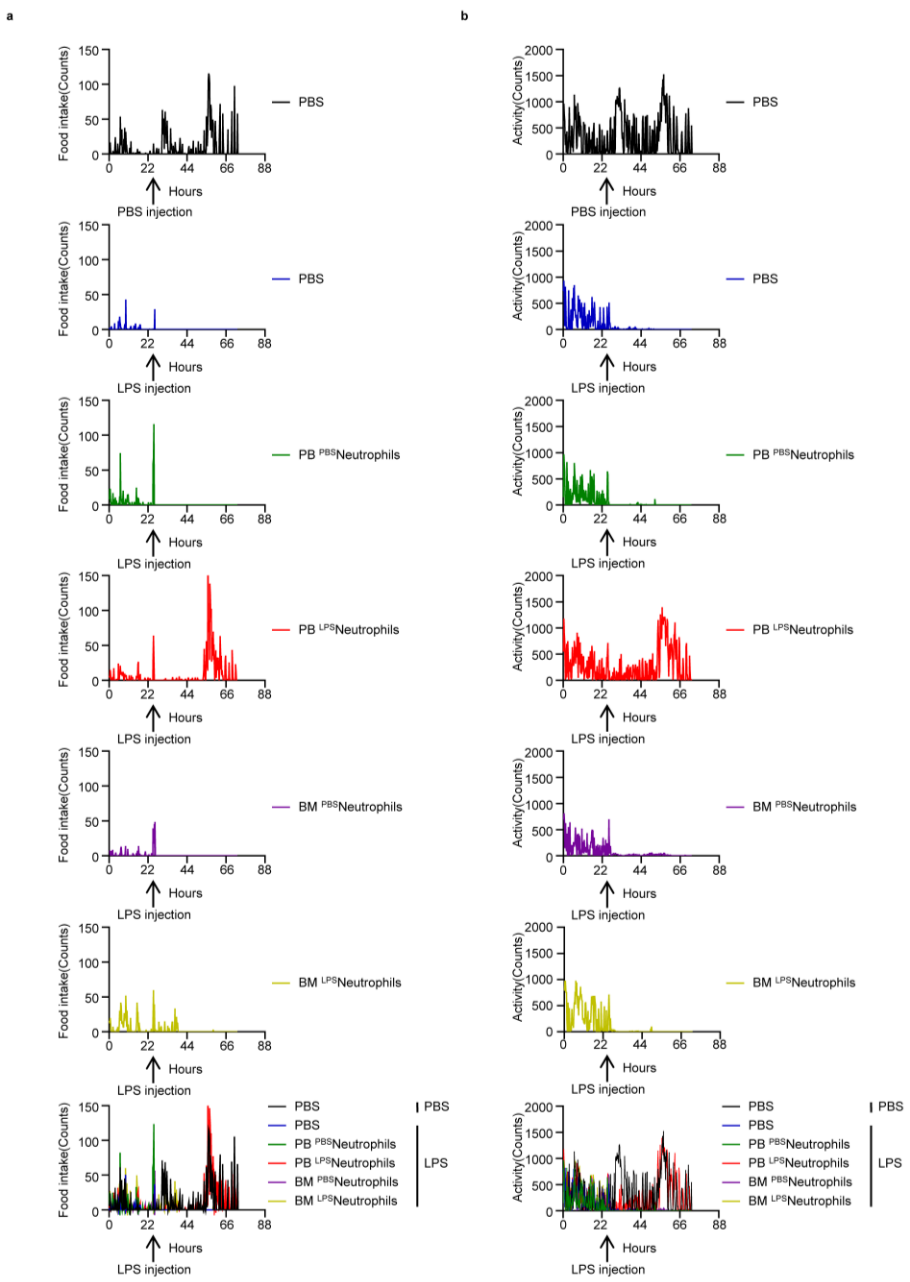

Supplementary Figure 3. LPS-primed circulating neutrophils improve food intake of the recipient mice under lethal LPS.

(**a-b**) Food intake (**a**) or spontaneous locomotor activity (**b**) of indicated recipient mice after PBS or lethal LPS challenge measured in metabolic cage.

Source data are provided as a Source Data file.

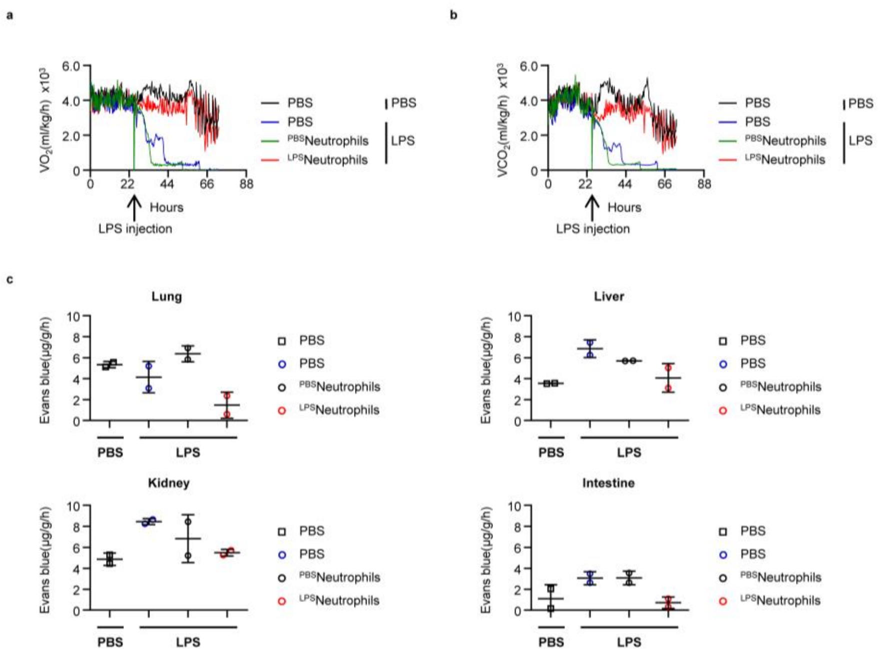

Supplementary Figure 4. LPS-primed circulating neutrophils rescue oxygen consumption, as well as vascular permeability of the recipients under lethal LPS. (**a-b**) Oxygen consumption rate (VO_2) or Carbon dioxide release rate (VCO_2) of the indicated recipient mice after PBS or lethal LPS challenge measured in metabolic cage. (**c**) Extravasation of Evans blue dye (EB) extracted from lung, liver, kidney and intestine 16 hours after PBS or lethal LPS (n = 2 per group).

Source data are provided as a Source Data file. Data are mean ± *SD*.

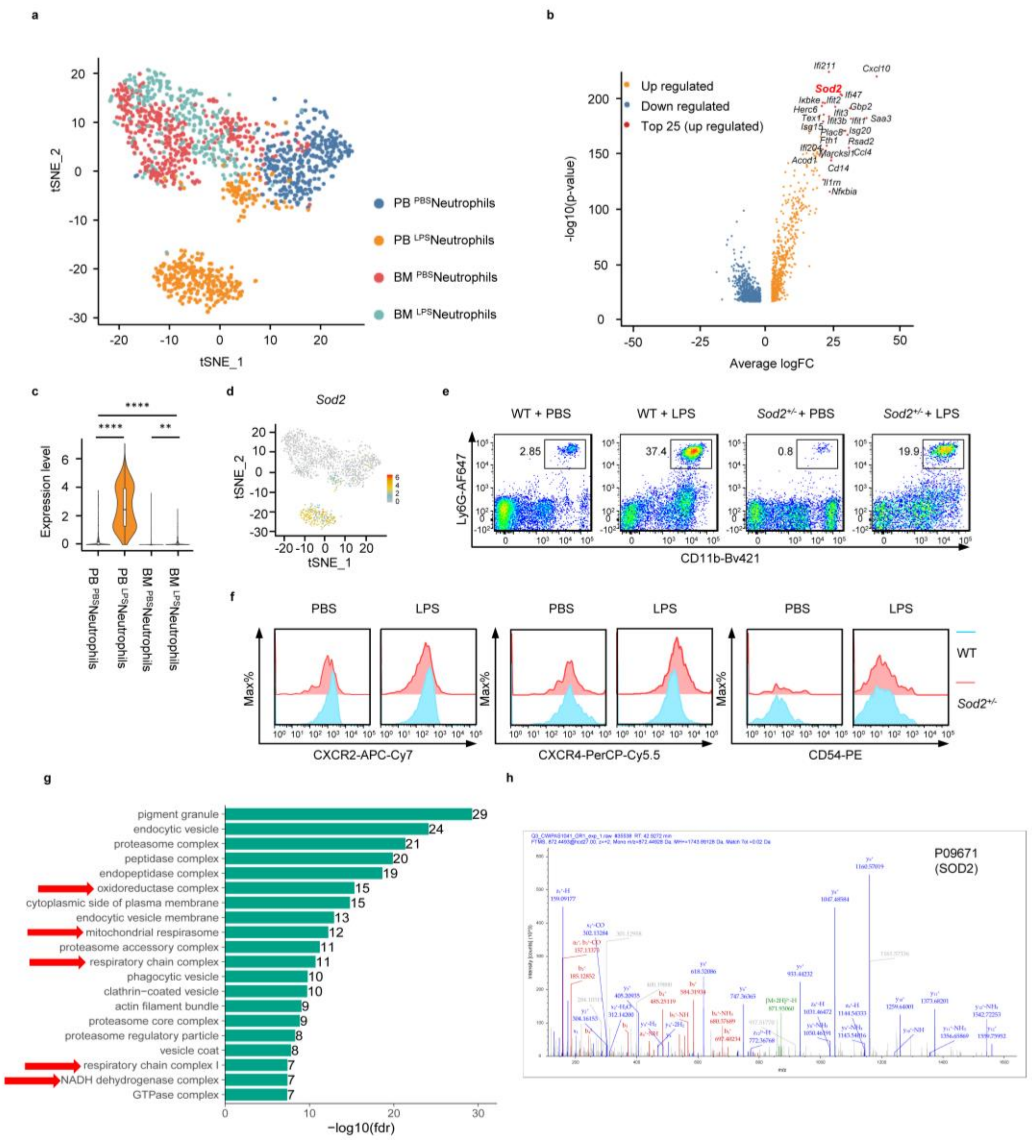

Supplementary Figure 5. *Sod2* is highly expressed by circulating neutrophils in response to LPS.

(**a**) T-distributed stochastic neighbor embedding (t-SNE) plot of LPS primed PB neutrophils (n = 363), PBS primed PB neutrophils (n = 348), LPS primed BM neutrophils (n = 271), PBS primed BM neutrophils (n = 439).

(**b**) Volcano plot depicting DEGs in LPS primed PB neutrophils (PB ^{LPS}neutrophils) versus PBS primed PB neutrophils (PB ^{PBS}neutrophils), LPS primed BM neutrophils (BM ^{LPS}neutrophils) and PBS primed BM neutrophils (BM ^{PBS}neutrophils).

(**c**) Expression of *Sod2* shown on t-SEN plot.

(**d**) Flow plots showing the expression of CD11b versus Ly6G among CD45^{+} cells in *Sod2*$^{+/-}$ mice and WT littermates 4 h after LPS.

(**e**) Flow analysis of the expression of CXCR2, CXCR4 and CD54 in *Sod2*$^{+/-}$ mice and

WT littermates 4 hours after LPS.

(**f**)Flow analysis of the expression of CXCR2, CXCR4 and CD54 in *Sod2*$^{+/-}$ mice and WT littermates 4 hours after LPS.

(**g**) GO analysis of the proteomic components of the ^{LPS}EVs. Red arrows indicate mitochondrion-associated clusters.

(**h**) Identification of Sod2 in the ^{LPS}EVs by mass spectrometry.

Two-tailed wilcoxon tests were used for statistical analyses. $^{**}p < 0.01$, $^{****}p < 0.0001$.

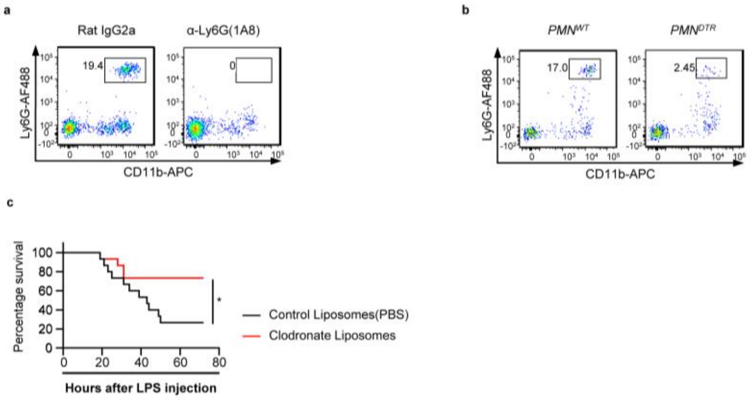

Supplementary Figure 6. Neutrophil depletion efficiencies by antibody or DT.

(**a-b**) Flow analysis of neutrophil depletion by antibody (**a**) or neutrophils ablation by DT (**b**).

(**c**) Survival of clodronate liposomes (n = 15), or control liposome(PBS) (n = 15) treated mice after lethal LPS. p =0.0218.

Source data are provided as a Source Data file. Data are mean ± *SD*. Log-rank (Mantel–Cox) test was used statistical analyses. *$p < 0.05$.
