# Supplementary material for: Neutrophils restrain sepsis associated coagulopathy via extracellular vesicles carrying superoxide dismutase 2 in a murine model of lipopolysaccharide induced sepsis
Source: Nat Commun. 2022 Aug 6;13:4583. doi: 10.1038/s41467-022-32325-w (PMC9357088; doi:10.1038/s41467-022-32325-w)
Supplement: Supplementary file 3 — Description of Additional Supplementary Files [file 41467_2022_32325_MOESM3_ESM.docx]

**Description of Additional Supplementary Files**

File Name: **Supplementary Movie 1.**

**LPS-primed circulating neutrophils rescue recipient mice from developing septic symptoms under lethal LPS challenge.**

Real-time recording of mouse activity 16 hours after PBS or lethal LPS injection. The mouse received PBS only was marked as No. 1; Mice were pre-injected with PBS-primed neutrophils (No. 2), LPS-primed neutrophils (No. 3) or PBS only (No. 4) followed by lethal LPS.

File Name: **Supplementary Movie 2**.

**LPS-primed circulating neutrophils improve blood perfusion within liver microcirculation under lethal LPS challenge, related to Figure 2.**

Real-time intravital imaging and recording of DiD-labeled erythrocyte (DiD-Red) trajectory within liver microcirculation (Dextran-Green) 1 hour after PBS. Scale bar, 100 μm.

File Name: **Supplementary Movie 3**.

**LPS-primed circulating neutrophils improve blood perfusion within liver microcirculation under lethal LPS challenge, related to Figure 2.**

Real-time intravital imaging and recording of DiD-labeled erythrocyte (DiD-Red) trajectory within liver microcirculation (Dextran-Green) 1 hour after lethal LPS. The mouse was pre-transferred with PBS. Scale bar, 100 μm.

File Name: **Supplementary Movie 4**.

**LPS-primed circulating neutrophils improve blood perfusion within liver microcirculation under lethal LPS challenge, related to Figure 2.**

Real-time intravital imaging and recording of DiD-labeled erythrocyte (DiD-Red) trajectory within liver microcirculation (Dextran-Green) 1 hour after lethal LPS. The mouse was pre-transferred with LPS-primed blood neutrophils. Scale bar, 100 μm.

File Name: **Supplementary Movie 5.**

**LPS-primed circulating neutrophils improve blood perfusion within liver microcirculation under lethal LPS challenge, related to Figure 2.**

Real-time intravital imaging and recording of DiD-labeled erythrocyte (DiD-Red) trajectory within liver microcirculation (Dextran-Green) 1 hour after lethal LPS. The mouse was pretreated with argatroban. Scale bar, 100 μm.

File Name: **Supplementary Movie 6.**

**Circulating neutrophils release EVs during migration, related to Figure 5.**

Real-time intravital imaging captured the release of vesicles by neutrophils (Ly6G-Red) within hepatic vessels (Dextran-Green). Scale bar, 10 μm.

File Name: **Supplementary Movie 7.**

**Circulating neutrophils release mitochondrion-containing EVs during migration, related to Figure 5.**

Real-time intravital imaging captured the release of mitochondrion-containing (Dendra2-Green) vesicles by neutrophils (Ly6G-Red) within hepatic vessels (CD144-Blue) using *PhAM^floxed^* **×** *Mrp8-Cre* mice. Scale bar, 7 μm.
