# Supplementary material for: Neutrophils restrain sepsis associated coagulopathy via extracellular vesicles carrying superoxide dismutase 2 in a murine model of lipopolysaccharide induced sepsis
Source: Nat Commun. 2022 Aug 6;13:4583. doi: 10.1038/s41467-022-32325-w (PMC9357088; doi:10.1038/s41467-022-32325-w)
Supplement: Supplementary file 11 — Reporting Summary [file 41467_2022_32325_MOESM11_ESM.pdf]

nature portfolio

Corresponding author(s): Xiaolong Liu

Last updated by author(s): Jun 26, 2022

Reporting Summary

Nature Portfolio wishes to improve the reproducibility of the work that we publish. This form provides structure for consistency and transparency in reporting. For further information on Nature Portfolio policies, see our Editorial Policies and the Editorial Policy Checklist.

Statistics

For all statistical analyses, confirm that the following items are present in the figure legend, table legend, main text, or Methods section.

n/a	Confirmed	
☐	☒	The exact sample size (*n*) for each experimental group/condition, given as a discrete number and unit of measurement
☐	☒	A statement on whether measurements were taken from distinct samples or whether the same sample was measured repeatedly
☐	☒	The statistical test(s) used AND whether they are one- or two-sided<br>*Only common tests should be described solely by name; describe more complex techniques in the Methods section.*
☒	☐	A description of all covariates tested
☒	☐	A description of any assumptions or corrections, such as tests of normality and adjustment for multiple comparisons
☐	☒	A full description of the statistical parameters including central tendency (e.g. means) or other basic estimates (e.g. regression coefficient) AND variation (e.g. standard deviation) or associated estimates of uncertainty (e.g. confidence intervals)
☐	☒	For null hypothesis testing, the test statistic (e.g. *F*, *t*, *r*) with confidence intervals, effect sizes, degrees of freedom and *P* value noted<br>*Give P values as exact values whenever suitable.*
☒	☐	For Bayesian analysis, information on the choice of priors and Markov chain Monte Carlo settings
☒	☐	For hierarchical and complex designs, identification of the appropriate level for tests and full reporting of outcomes
☒	☐	Estimates of effect sizes (e.g. Cohen's *d*, Pearson's *r*), indicating how they were calculated

Our web collection on statistics for biologists contains articles on many of the points above.

Software and code

Policy information about availability of computer code

Data collection	Flow cytometry: BD FACSDiva v8.0.2<br>Microscopy: Olympus FLUOVIEW FV31S-SW v2.3.1.163<br>Real Time PCR: LightCycler 96 SW v1.1<br>TEM: FEI Tecnai V4.0.3<br>Mass spectra:Proteome Discoverer v2.4
Data analysis	Statistics: GraphPad Prism v6.0<br>Image Analysis: Imaris v9.5<br>Flow Cytometry: FlowJo v10<br>Single cell sequencing Analysis: Cutadapt (v1.15), STAR (v2.6.0a), HTSeq (v0.11.2), Seurat (v3.2.3)

For manuscripts utilizing custom algorithms or software that are central to the research but not yet described in published literature, software must be made available to editors and reviewers. We strongly encourage code deposition in a community repository (e.g. GitHub). See the Nature Portfolio guidelines for submitting code & software for further information.

Data

Policy information about availability of data

All manuscripts must include a data availability statement. This statement should provide the following information, where applicable:

- Accession codes, unique identifiers, or web links for publicly available datasets
- A description of any restrictions on data availability
- For clinical datasets or third party data, please ensure that the statement adheres to our policy

Source Data are provided with this paper. Mouse reference genome mm9 Murine (https://www.ncbi.nlm.nih.gov/assembly/GCF_000001635.18/) and Protein Database: UniProt release 2022_01 (https://www.uniprot.org/uniprot/?query=*&fil=organism%3A%22Mus+musculus+%28Mouse%29+%5B10090%5D%22+AND+reviewed%3Ayes) are used.The raw sequencing data and processed data for single-cell RNA-seq generated in this study have been deposited in the NCBI GEO database under accession code GSE206825 (https://www.ncbi.nlm.nih.gov/geo/query/acc.cgi?acc=GSE206825). Proteomic results are provided in the Source Data file.

Field-specific reporting

Please select the one below that is the best fit for your research. If you are not sure, read the appropriate sections before making your selection.

[x] Life sciences   [ ] Behavioural & social sciences   [ ] Ecological, evolutionary & environmental sciences

For a reference copy of the document with all sections, see nature.com/documents/nr-reporting-summary-flat.pdf

Life sciences study design

All studies must disclose on these points even when the disclosure is negative.

Sample size	No power analysis were used to predetermine sample size. Experimental group sizes of at least 3 animals were used.
Data exclusions	No data was exclued from analysis.
Replication	All experimental findings described in this manuscript were reliably reproduced. All data were pooled from at least two independent experiments.
Randomization	Animals were allocated into groups based on donor cell or genotypes. Gender-matched litter-mates from independent cohorts were used whenever possible. For experiments other than mice studies, samples/participants were allocated into experimental groups randomly.
Blinding	Researchers were blinded whenever possible. Flow samples were identified by ID numbers during collection and Flowjo analysis. Genotypes were revealed during grouping and comparing the collected data. Image collection by microscopy was performed while blinded to the animals' genotype and animal treatment condition.

Reporting for specific materials, systems and methods

We require information from authors about some types of materials, experimental systems and methods used in many studies. Here, indicate whether each material, system or method listed is relevant to your study. If you are not sure if a list item applies to your research, read the appropriate section before selecting a response.

Materials & experimental systems

n/a	Involved in the study	
[ ]	[x]	Antibodies
[x]	[ ]	Eukaryotic cell lines
[x]	[ ]	Palaeontology and archaeology
[ ]	[x]	Animals and other organisms
[ ]	[x]	Human research participants
[x]	[ ]	Clinical data
[x]	[ ]	Dual use research of concern

Methods

n/a	Involved in the study	
[x]	[ ]	ChIP-seq
[ ]	[x]	Flow cytometry
[x]	[ ]	MRI-based neuroimaging

Antibodies

Antibodies used	Anti-mouse CD45-Brilliant Violet 421 BioLegend Cat#103133;Lot#B294378<br>Anti-mouse F4/80-PE Invitrogen Cat#12-4801-82;Lot#2049425<br>Anti-Mouse CD16/CD32 BD Biosciences Cat#553141;Lot#9060742

Anti-CD11b-APC Invitrogen Cat#17-0112-82;RRID:Lot#2010662
Anti-mouse Ly-6G-Alexa Fluor 488 BioLegend Cat#127626;Lot#B312093
Anti-mouse Ly-6G-Alexa Fluor 647 BioLegend Cat#127610;
Anti-Fibrin Sigma-Aldrich Cat#MABS2155;Lot#3668174
Anti-mouse CD31-Alexa Fluor 647 BioLegend Cat#102416;Lot#B332763
Anti-mouse CD31-PE/Cyanine7 BioLegend Cat#102417;Lot#B312606
Anti-SOD2/MnSOD abcam Cat#ab68155;Lot#GR258733-31
Anti-rabbit β-Actin-HRP Beyotime Cat#AF5006;
Anti-rabbit IgG-HRP Cell Signaling Technology Cat#7074S;Lot#29
Anti-Ly6G Cell Signaling Technology Cat#87048S:Lot#1
Anti-COX IV Cell Signaling Technology Cat#4844S;Lot#3
Anti-mouse CD45 FITC BioLegend Cat#103108;RRID:Lot#B289551
Anti-mouse/human CD11b-Brilliant Violet 421 BioLegend Cat#101236;Lot#B336198
Anti-mouse CD184 (CXCR4)-PerCP/Cyanine5.5 BioLegend Cat#146510;RRID:Lot#B291283
Anti-mouse CD182 (CXCR2)-APC/Cyanine7 BioLegend Cat#149314;Lot#B286705
Anti-CD54 (ICAM-1)-PE Invitrogen Cat#12-0542-82;Lot#4299996
InVivoMAb anti-mouse Ly6G Bio X Cell Cat#BE0075-1;Lot#737721M1
InVivoMAb rat IgG2a isotype control Bio X Cell Cat#BE0089;Lot#796721M1
Anti-mouse Ly6G-PE BD Biosciences Cat#551461;Lot#9099746
Anti-CD144 (VE-cadherin)-eFluor 660 Invitrogen Cat#50-1441-82;Lot#2289519
Donkey anti-rabbit IgG (H+L)-Cy3 Jackson ImmunoResearch Cat#711-165-152;
Donkey anti-mouse IgG (H+L)-Cy3 Jackson ImmunoResearch Cat#715-165-150;
Anti-human TOMM22 Cat#66562-1-Ig;Lot#10005339
Anti-mouse/human CD15 Cat#ab135377;Lot#GR3395417-3

Validation

All antibodies were validated by the manufacturer. Lot validation and quality control is available from the commercial source using the lot numbers listed.
Anti-mouse CD45-Brilliant Violet 421 BioLegend Cat#103133;Species reactivity:mouse;Tested applications:Flow cytometry.SB
Anti-mouse F4/80-PE Invitrogen Cat#12-4801-82;Species reactivity:mouse;Tested applications:IHC,IHC(F), IF,Misc,flow cytometry
Anti-Mouse CD16/CD32 BD Biosciences Cat#553141;Species reactivity:mouse;Tested applications:Blocking,IHC(F), IP,flow cytometry
Anti-CD11b-APC Invitrogen Cat#17-0112-82;Species reactivity:mouse;Tested applications:IHC(F), IHC, IF,flow
Anti-mouse Ly-6G-Alexa Fluor 488 BioLegend Cat#127626;Species reactivity:mouse;Tested applications:Flow cytometry,IHC-F,SB
Anti-mouse Ly-6G-Alexa Fluor 647 BioLegend Cat#127610;Species reactivity:mouse;Tested applications:Flow cytometry,IHC-F
Anti-Fibrin Sigma-Aldrich Cat#MABS2155;Species reactivity:Mouse, Human;Tested applications: Immunofluorescence, Immunohistochemistry, Peptide Inhibition Assay, Radioimmunoassay, and Western Blotting.
Anti-mouse CD31-Alexa Fluor 647 BioLegend Cat#102416;Species reactivity:mouse;Tested applications:Flow cytometry
Anti-mouse CD31-PE/Cyanine7 BioLegend Cat#102417;Species reactivity:mouse;Tested applications:Flow cytometry
Anti-SOD2/MnSOD abcam Cat#ab68155;Species reactivity:Mouse, Rat, Human; Tested applications:WB, IHC-P
Anti-Ly6G Cell Signaling Technology Cat#87048S;Species reactivity:Mouse;Tested applications:WB,IHC
Anti-COX IV Cell Signaling Technology Cat#4844S;Species reactivity:Mouse, Human;Tested applications:WB,IP,IHC
Anti-mouse CD45 FITC BioLegend Cat#103108;Species reactivity:mouse;Tested applications:Flow Cytometry
Anti-mouse/human CD11b-Brilliant Violet 421 BioLegend Cat#101236;Species reactivity:Mouse, Human;Tested applications:Flow Cyt
Anti-mouse CD184 (CXCR4)-PerCP/Cyanine5.5 BioLegend Cat#146510;Species reactivity:mouse;Tested applications:Flow Cytometry
Anti-mouse CD182 (CXCR2)-APC/Cyanine7 BioLegend Cat#149314;Species reactivity:mouse;Tested applications:Flow Cytometry
Anti-CD54 (ICAM-1)-PE Invitrogen Cat#12-0542-82;Species reactivity:mouse;Tested applications:Flow Cytometry
InVivoMAb anti-mouse Ly6G Bio X Cell Cat#BE0075-1;Species reactivity:mouse;Applications:in vivo neutrophil depletion,in vivo MDSC depletion,Immunofluorescence,Immunohistochemistry (paraffin),Immunohistochemistry (frozen),Flow cytometry
Anti-mouse Ly6G-PE BD Biosciences Cat#551461;Species reactivity:mouse;Tested applications:Flow cytometry
Anti-CD144 (VE-cadherin)-eFluor 660 Invitrogen Cat#50-1441-82;Species reactivity:mouse;Tested applications:WB, IHC, IF,flow,ChIP
Anti-human TOMM22 proteintech Cat#66562-1-Ig;Species reactivity:Human;Tested applications:WB, IHC, IF
Anti-mouse/human CD15 abcam Cat#ab135377;Species reactivity:Mouse, Human;Tested applications:Flow Cyt, IHC-P

Animals and other organisms

Policy information about studies involving animals; ARRIVE guidelines recommended for reporting animal research

Laboratory animals

All mice were of the C57BL/6J background. 7- to 12-week-old male mice were used.
Mice purchased from The Jackson Laboratory:
B6.Cg-Tg (S100A8-cre,-EGFP) 1Ilw/J
B6; 129S-Gt(ROSA)26Sortm1(CAG-COX8A/Dendra2)Dcc /J

Mice purchased from GemPharmatech Company:
B6/JGpt-Sod2em18Cd19076 /Gpt

Mice provide by Dr. Wen-Biao Gan at Peking University, Shenzhen Graduate School:
C57BL/6-Gt(ROSA)26Sortm1(HBEGF)Awai /J

Mice provide by Dr. Li Yu at Tsinghua University:
TSPAN9-/-

Wild animals	No wild animals were used in the study.
Field-collected samples	No field collected samples were used in the study
Ethics oversight	All experimental animal procedures were approved by Institutional Animal Care and Use Committees (IACUCs) of Center for Excellence in Molecular Cell Science, CAS.

Note that full information on the approval of the study protocol must also be provided in the manuscript.

Human research participants

Policy information about studies involving human research participants

Population characteristics	In this manuscript, a total of 5 blood samples were obtained from healthy donors with age range of 22-29 and sex distribution of 2 females/3 males.
Recruitment	Blood samples were collected from healthy donors. The participants were recruited in Center for Excellence in Molecular Cell Science, Chinese Academy of Sciences and surroundings by means of posters and flyers. Investigators were blinded to donors' information.
Ethics oversight	The study has been approved by the Institutional Review board of Center for Excellence in Molecular Cell Science, Chinese Academy of Sciences. Informed consent was received from each donor.

Note that full information on the approval of the study protocol must also be provided in the manuscript.

Flow Cytometry

Plots

Confirm that:

- [x] The axis labels state the marker and fluorochrome used (e.g. CD4-FITC).
- [x] The axis scales are clearly visible. Include numbers along axes only for bottom left plot of group (a 'group' is an analysis of identical markers).
- [x] All plots are contour plots with outliers or pseudocolor plots.
- [x] A numerical value for number of cells or percentage (with statistics) is provided.

Methodology

Sample preparation	For liver endothelial cell preparation, liver was digested by a two-step liver collagenase perfusion. After digestion, the liver was gently extruded in a dish containing 15 ml of dulbecco's modified eagle medium (DMEM). Single cells released from the liver were collected and filtered through a 100μm cell strainer. The single cell suspension was centrifuged at 50 g for 2 minutes. The pelleted hepatocytes were discarded. The supernatant was collected for a further centrifugation at 500g for 7 minutes to pellet immune cells and endothelial cells. The obtained cells were washed , stained and analyzed by flow cytometry.
Instrument	Flow data were collected on BD LSRFortessa II
Software	Flow data were collected using BD FACSDiva , and analyzed using Flowjo v10.
Cell population abundance	Blood and BM neutrophils were isolated by EasySep Mouse Neutrophil Enrichment Kit (Stem Cell). The isolated neutrophils were washed twice with PBS and maintained on ice for further experiments.
Gating strategy	All cells were gated on FSC/SSC, singlets and live cells.<br>Liver endothelial cell: CD45-/CD31+<br>Neutrophils: CD45+/CD11b+/Ly6G+

- [ ] Tick this box to confirm that a figure exemplifying the gating strategy is provided in the Supplementary Information.
